# 

**Published:** 1893-10

**Authors:** 


CONTENTS—OCTOBER, 1893.—VOL. IX., NO. 4.

ORIGINAL COMMUNICATIONS—

Tubercular Meningitis, a case of. Prof. A. J. Smith, M. D., Galveston . . . 147
Congenital Anhyclosis of Sixteen Joints. Report of a Case. S. E. Milliken, M. D., New York . . . 154
Teaching Anatomy in Medical Schools. B. T. Flavin, M. D., Demonstrator, Texas Medical College, Galveston . . . 155
Polio-Myilitis. J. T. Eskridge, M. D., Denver, Colorado. . . . 160

ABSTRACTS OF CURRENT MEDICAL LITERATURE—

Department of Dermatology. Dr. Isadore Dyer, New Orleans . . . 169

CORRESPONDENCE—

Some Cranial Surgery. T. J. Pugh, M. D., Hearne, Tex . . . 170

EDITORIAL—

What's in a Name? . . . 173
Our Wile(y) Friend . . . 176
The Texas Medical College—Third Session . . . 178

MEDICAL NEWS AND MISCELLANY—

The Wm. F. Jenks Memorial Prize . . . 172
Our Esteemed Contemporary, H. H. Haralson . . . 182
The Doctor's Lament . . . 183
The Country Doctor . . . 184

BOOK NOTICES—

A System of Genito-Urinary Diseases, Syphilology and Dermatology. By Prince A. Morrow, M. D . . . 187
Brain Surgery. M. Allen Starr, M. D . . . 189
Hand Book of Local Therapeutics . . . 196
Diseases of Inebriety. Carothers . . . 191
Medical Consultation Book, Hachenberg . . . 192
Dunglison's New Pronouncing Dictionary . . . 192

PUBLISHERS' NOTES . . . 193

